# Genome-wide identification of the peptide transporter family in rice and analysis of the PTR expression modulation in two near-isogenic lines with different nitrogen use efficiency

**DOI:** 10.1186/s12870-020-02419-y

**Published:** 2020-05-06

**Authors:** Xinghai Yang, Xiuzhong Xia, Yu Zeng, Baoxuan Nong, Zongqiong Zhang, Yanyan Wu, Qinglan Tian, Weiying Zeng, Ju Gao, Weiyong Zhou, Haifu Liang, Danting Li, Guofu Deng

**Affiliations:** 1 Rice Research Institute, Guangxi Academy of Agricultural Sciences, 174 East Daxue Road, Nanning, 530007 Guangxi China; 2 Biotechnology Research Institute, Guangxi Academy of Agricultural Sciences, Nanning, 530007 Guangxi China; 3 Cash Crops Research Institute, Guangxi Academy of Agricultural Sciences, Nanning, 530007 Guangxi China; 4 Guangxi Crop Genetic Improvement and Biotechnology Laboratory, Guangxi Academy of Agricultural Sciences, Nanning, 530007 Guangxi China

**Keywords:** Rice, PTR family, Phylogenetic analysis, Expression profile, Gene regulatory network

## Abstract

**Background:**

Nitrogen (N) is a major nutrient element for crop growth. In plants, the members of the peptide transporter (PTR) gene family may involve in nitrate uptake and transport. Here, we identified PTR gene family in rice and analyzed their expression profile in near-isogenic lines.

**Results:**

We identified 96, 85 and 78 PTR genes in Nipponbare, R498 and *Oryza glaberrima*, and the phylogenetic trees were similar in Asian cultivated rice and African cultivated rice. The number of PTR genes was higher in peanut (125) and soybean (127). The 521 PTR genes in rice, maize, sorghum, peanut, soybean and *Arabidopsis* could be classified into 4 groups, and their distribution was different between monocots and dicots. In Nipponbare genome, the 25 PTR genes were distributed in 5 segmental duplication regions on chromosome 1, 2, 3, 4, 5, 7, 8, 9, and 10. The PTR genes in rice have 0–11 introns and 1–12 exons, and 16 of them have the NPF (NRT1/PTR family) domain. The results of RNA-seq showed that the number of differentially expressed genes (DEGs) between NIL15 and NIL19 at three stages were 928, 1467, and 1586, respectively. Under low N conditions, the number of differentially expressed PTR genes increased significantly. The RNA-seq data was analyzed using WGCNA to predict the potential interaction between genes. We classified the genes with similar expression pattern into one module, and obtained 25 target modules. Among these modules, three modules may be involved in rice N uptake and utilization, especially the brown module, in which hub genes were annotated as protein kinase that may regulate rice N metabolism.

**Conclusions:**

In this study, we comprehensively analyzed the PTR gene family in rice. 96 PTR genes were identified in Nippobare genome and 25 of them were located on five large segmental duplication regions. The Ka/Ks ratio indicated that many PTR genes had undergone positive selection. The RNA-seq results showed that many PTR genes were involved in rice nitrogen use efficiency (NUE), and protein kinases might play an important role in this process. These results provide a fundamental basis to improve the rice NUE via molecular breeding.

## Background

Rice is one of the most important food crops in the world. As the population continues to grow, the food security issues are becoming more and more prominent [1]. The application of N fertilizer has been the main method to improve rice production, but the excessive use of N fertilizer also increases the production costs, and causes serious damage to environment. As the concept of “green super rice” has been proposed [2], developing the rice varieties with more efficient use of N fertilizers have become the key to improving agricultural production while preserving the environment.

NRT1s play important roles in nitrate uptake and transport in rice [3, 4]. NRT1 family mainly includes low-affinity nitrate transporters and belongs to PTR family. Thus, NRT1/PTR family is also named as NPF. The major NUE-related PTR genes have been identified in rice [5]. *OsNRT1*is the first PTR gene identified in rice [6] and is located at the same locus as *OsNPF8.9* [7]; *OsNRT1* has two transcripts, *OsNRT1.1a* and *OsNRT1.1b*. *OsNRT1.1b* encodes a PTR protein with 6 transmembrane domains, and its overexpression can increase rice N uptake under high or low N conditions, while *OsNRT1.1a* only works under high N conditions [8]. Based on the homology with *AtNRT1.1*, Plett et al. [9] named the three homologous genes in rice as *OsNRT1.1A*, *OsNRT1.1B*, and *OsNRT1.1C.* The overexpression of *OsNRT1.1A* can up-regulate the genes related to nitrate and ammonium transport [10]. Hu et al. [11] found that *LOC_Os10g40600* encoded a nitrate transporter NRT1.1B, which could affect the NUE of *Indica* and *Japonica* via regulating rice root microbial population and altering the rhizosphere microenvironment [12]. *OsNPF2.2* can unload nitrate from the rice xylem and participate in nitrate transport from root to stem, thus affecting the growth and development of vascular system, or even the whole plant [13]. Overexpression of *OsPTR6* increases the expression of ammonium transporter gene and activates glutamine synthetase, which effects can promote rice growth, but reduces NUE under high ammonium conditions [14]. Fang et al. [15] showed that altering the expression of *OsPTR9* affected NUE, plant growth and rice production. *OsNPF2.4* plays a role in NO_3_-absorption, long-distance transport and redistribution; also, altering its expression indirectly affects the reuse of potassium in roots and stems. Hu et al. [16] concluded that *OsNPF7.2* played a role in the intracellular distribution of nitrate in roots, and affected rice growth under high nitrate condition. Recently, Gao et al. [17] used the difference in NUE between *indica* and *japonica* rice to clone a gene encoding NADH / NADPH-dependent nitrate reductase OsNR2, which interacts with the nitrate transporter OsNRT1.1B to promote nitrate absorption by indica. Tang et al. [18] cloned a dual-affinity nitrate transporter gene *OsNPF6.1* and found that the transcription factor *OsNAC42* can activate *OsNPF6.1*, which then enhances N absorption and NUE in rice. In summary, these rice PTR genes may play an important role in NUE, and it’s likely that there are still other PTR genes in rice genome involved in N metabolism pathway.

In the previous study, we identified a NUE-related QTL *qNUE6* in rice, and *LOC_Os06g15370* may be the ideal candidate gene [19]. The annotation for *LOC_Os06g15370* is a peptide transporter, and we named this gene as *OsPTR10*, as *OsNPF3.1* [7]. Here, we use the latest Nipponbare genome to identify PTR genes, and compare them with the PTR genes in R498, *Oryza glaberrima*, maize, sorghum, peanut, soybean and *Arabidopsis*. The main aims of this study are: (i) determine the number of PTR genes in rice; (ii) understand the evolutionary relationship of PTR genes in rice and other 5 plants; (iii) analyze the regulatory network of PTR genes in rice NUE.

## Results

### Identification of PTR gene in cultivated rice

Among the 27 *Oryza* species, Asian cultivated rice (*Oryza sativa* L.) and African cultivated rice (*Oryza glaberrima* Steud*.*) are the two species that have been domesticated and utilized by humans [20]. Using the conserved region sequence of PTR gene family, 96 PTR genes were identified in Nipponbare (*Oryza sativa* L. ssp*. japonica*) (Table S2), 85 were identified in R498 (*Oryza sativa* L. ssp. *indica*) (Table S3), and 78 were identified in *Oryza glaberrima* (Table S4).

### Phylogenetic analysis of PTR genes

**Fig. 1:**
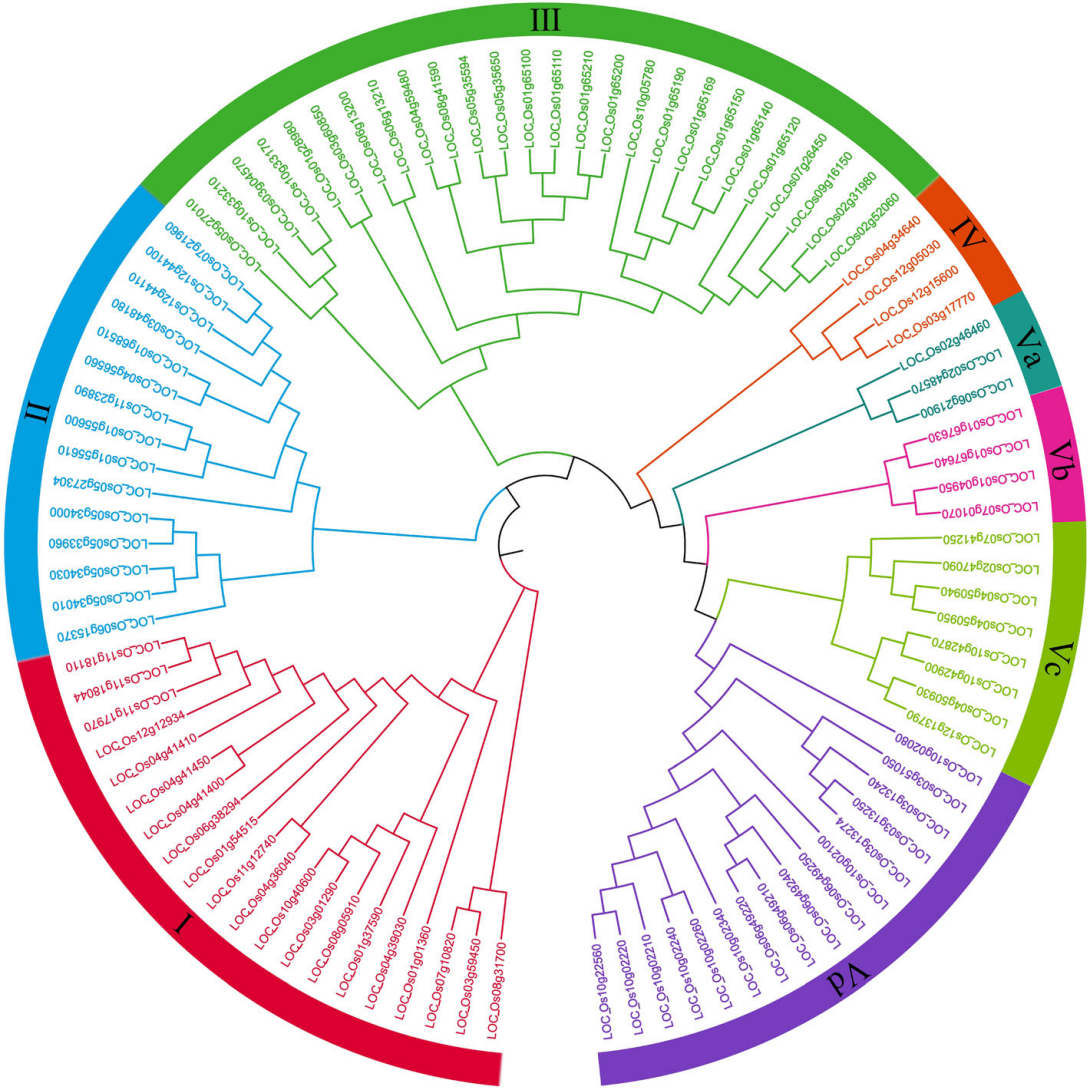
Phylogenetic tree and distribution of PTR genes in Nipponbare. The different-colored arcs indicate different groups. Group V classified into 4 subgroups. Group I, II, III, IVand V contain 20, 15, 26, 4 and 31 genes. Subgroup Va, Vb, Vc, and Vd contain 3, 4, 8 and 16 genes

Multi-sequence alignment and phylogenetic analysis were performed on the protein sequences of 96 rice PTR genes using MEGA. Based on the evolutionary relationship, the 96 PTR genes were classified into 5 groups, and groupIto V contained 20, 15, 26, 4 and 31 genes, respectively. The group V contained the most genes, which could be further classified into four subgroups, Va (3), Vb (4), Vc (8), and Vd (16) (Fig. 1).

**Fig. 2:**
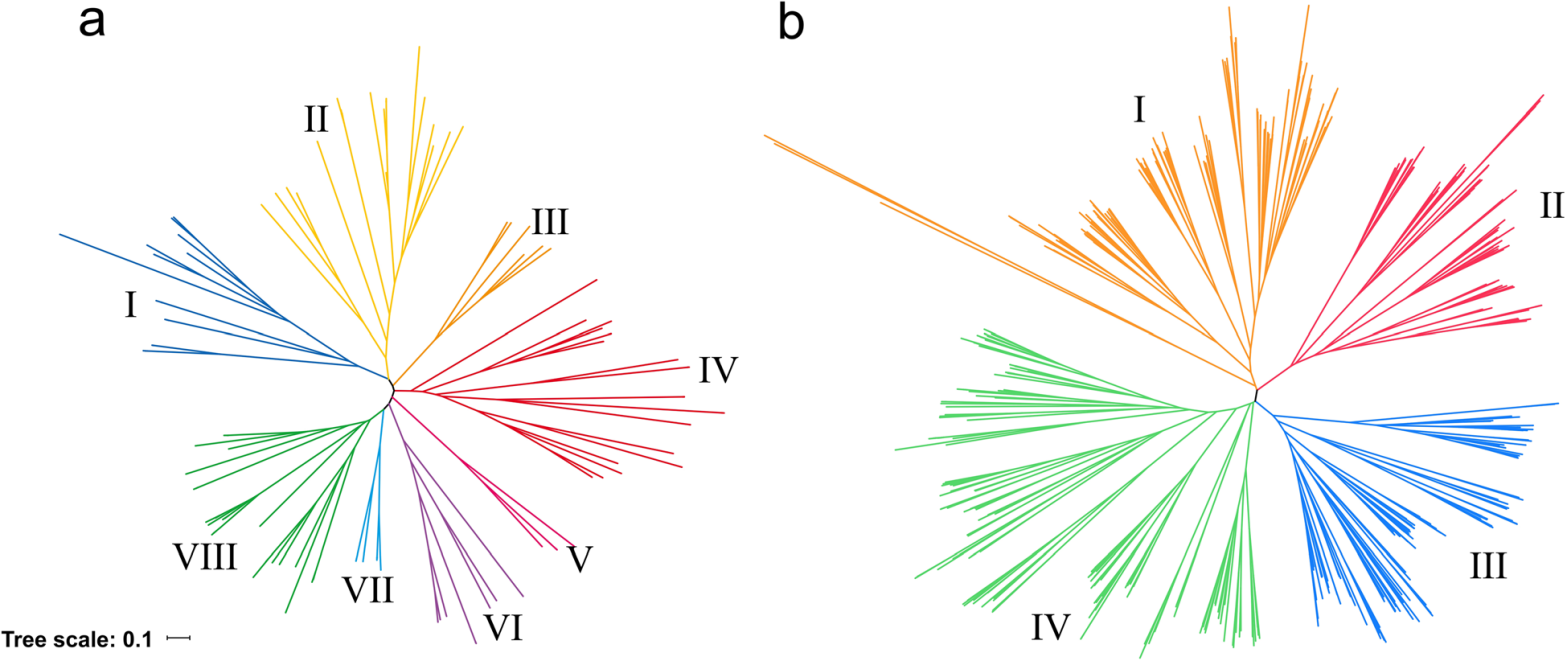
Distribution of PTR genes in 8 plant species. **a** Neighbor-joining tree of 259 PTR genes in Nipponbare, R498 and *Oryza glaberrima*, theese genes classified into 8 groups. The different-colored arcs indicate different groups. Group I, II, III, IV, V, VI, VI, VII andVII contain 29, 73, 17, 46, 15, 23, 11 and 45 genes. **b** Neighbor-joining tree of 543 PTR genes in rice, maize, sorghum, peanut, soybean and *Arabidopsis*, theese genes classified into 4 groups. Group I, II, III and IV contain 140, 87, 135 and 181 genes. Group III contains the most PTR genes of monocots, and group IV had the most PTR genes in dicots. The scale bar indicates the simple matching distance

To understand the evolutionary relationship of PTR genes between different *Oryza* species or subspecies, phylogenetic analysis was carried out using the PTR genes from Nipponbare, R498 and *Oryza glaberrima*. These PTR genes could be classified into 8 groups: group II contained 73 genes, which was the most, whereas group VII had only 11 genes (Fig. 2a). The distribution of PTR genes in the 8 groups were similar among the three *Oryza* species (Table S5). Nipponbare genome was used for the further analysis as shown below.

Leguminous plants can form a symbiotic relationship with N-fixing bacteria, which can convert N_2_ in the air into NH_3_. Thus, we analyzed the leguminous plants peanuts and soybeans. We also analyzed the dicotyledonous plant *Arabidopsis*, and the important monocotyledonous plants maize, sorghum and rice. The plant genome sizes of these six species were 2.5 Gb [21], 1.1 Gb [22], 125 Mb [23], 2.3 Gb [24], 730 Mb [25] and 466 Mb [26], respectively. Although the maize genome was large, it only contained 66 PTR genes, whereas the legume genome contained many more PTR genes: 125 for peanuts and 117 for soybeans. This might be related to the need of N transport after N fixation in legumes.

The phylogenetic tree was constructed based on the protein sequences of 543 PTR genes from the six species (Table S6), and these genes could be classified into 4 groups, I,II,III and IV (Fig. 2b). The PTR gene distribution in rice, maize and sorghum were similar, and group III contained the most monocots PTR genes. Similarly, the PTR gene distribution in peanut, soybean and *Arabidopsis* were similar, and group IV had the most dicots PTR genes.

### The chromosome location and segmental duplication events of rice PTR genes

**Fig. 3:**
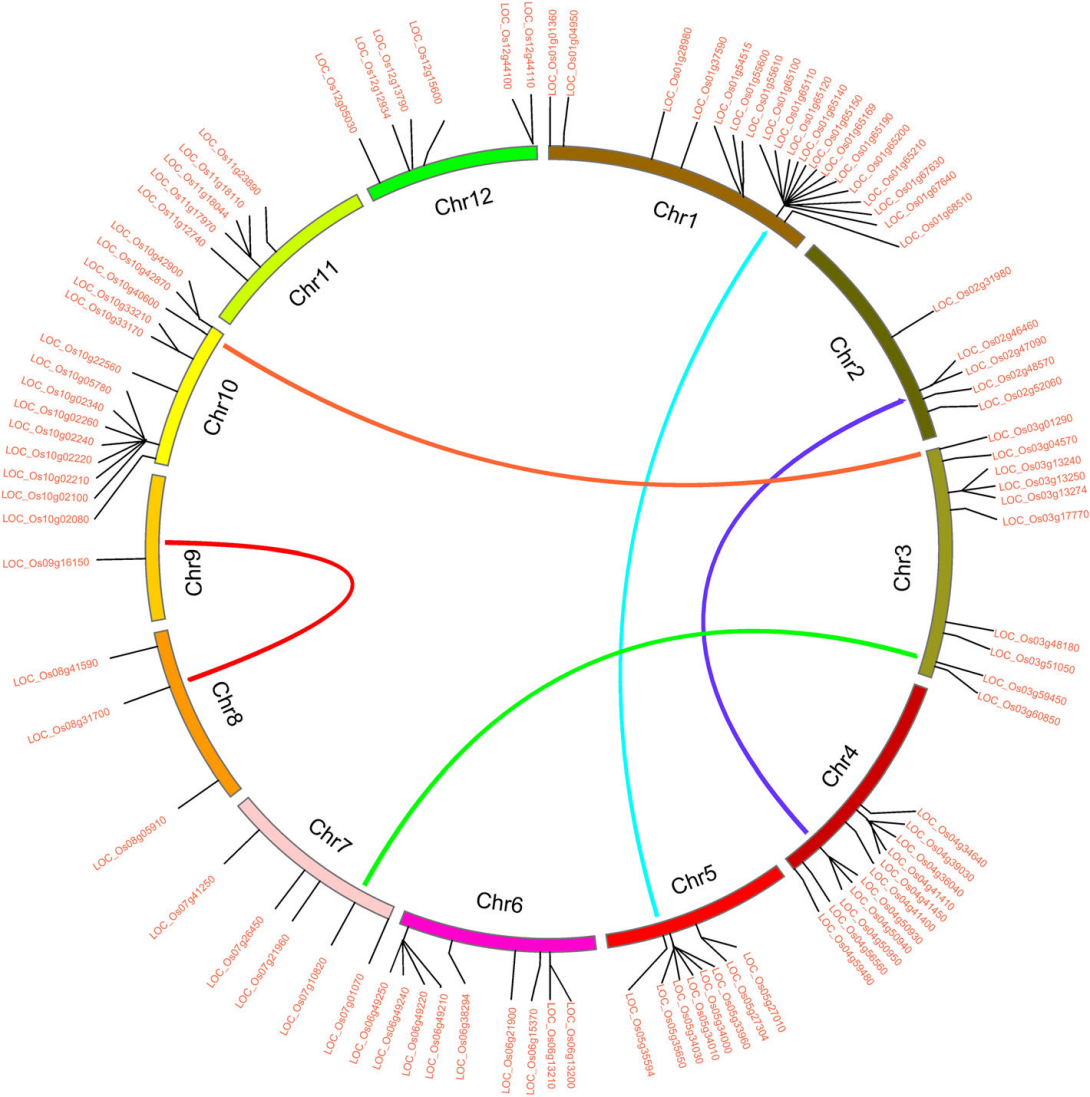
Schematic representations for the chromosomal distribution and segmental duplication events of rice PTR genes. The duplicated blocks were connected with clolored lines. The PTR genes tightly arranged together represent gene cluster. The different-colored arcs indicate different chromosomes. The chromosome number is indicated at the bottom of each chromosome

The 96 PTR genes were unevenly distributed on 12 chromosomes in rice. Chromosome 1 had the most PTR genes and chromosome 9 contained the least. The details are shown in Fig. 3. 59 PTR genes, accounting for 60.4% of the total PTR genes, formed 17 gene clusters. Except chromosome 7, 8, and 9, all other chromosomes contained PTR gene clusters, and the cluster in 37.79–37.83 Mb region of chromosome 1 was the biggest, containing 9 PTR genes.

The segmental duplication events were analyzed using the 96 PTR genes. The results showed that there were 5 large genomic duplication regions, which contained 25 PTR gens: (i) 37.76 Mb − 38.75 Mb on chromosome 1 corresponding to 20.31 Mb − 21.21 Mb on chromosome 5; (ii) 27.74 Mb − 28.95 Mb on chromosome 2 corresponding to 29.11 Mb − 30.42 Mb on chromosome 4; (iii) 0.15 Mb − 0.21 Mb on chromosome 3 corresponding to 21.76 Mb - 21.90 Mb on chromosome 10; (iv) 33.56 Mb − 33.92 Mb on chromosome 3 corresponding to 5.31 Mb − 6.14 Mb on chromosome 7; (v) 19.49 Mb − 19.744 Mb on chromosome 8 corresponding to 12.13 Mb − 12.45 Mb on chromosome 9 (Table 1).

**Table 1:** The duplicated PTR genes in the 5 duplicated blocks

Block	Copy 1 of a duplicated block				Copy 2 of a duplicated block			
	Chromosome	Interval (bp)	Length (bp)	Number of PTR genes	Chromosome	Interval (bp)	Length (bp)	Number of PTR genes
1	1	37,763,432-38,745,918	982,487	9	5	20,314,680-21,207,906	893,227	6
2	2	27,744,016-28,948,417	1,204,402	2	4	29,106,041–30,424,148	1,318,108	3
3	3	152,358-211,839	59,482	1	10	21,757,768-21,903,586	145,819	1
4	3	33,563,702-33,921,098	357,397	1	7	5,313,788-6,141,197	827,410	1
5	8	19,490,070-19,739,231	249,162	1	9	12,126,818-12,445,866	319,049	0

**Fig. 4:**
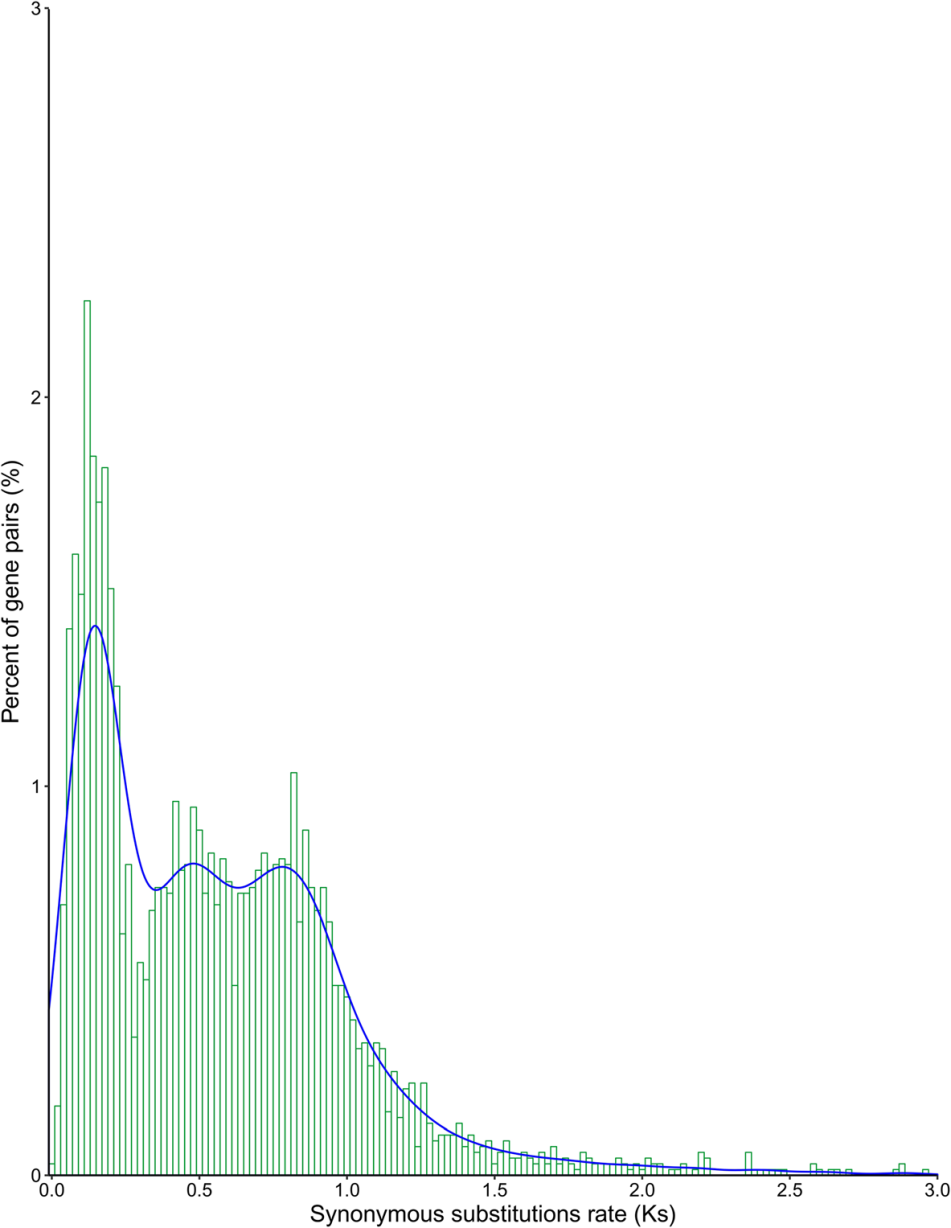
Whole genome duplications of rice using PTR genes. X-axis indicates the synonymous mutation rate Ks. Y-axis indicates PTR paralogous gene pairs. Evry peak represents a genome duplication event. The farther away from the origin represents the earlier the genome duplication event occurs

**Fig. 5:**
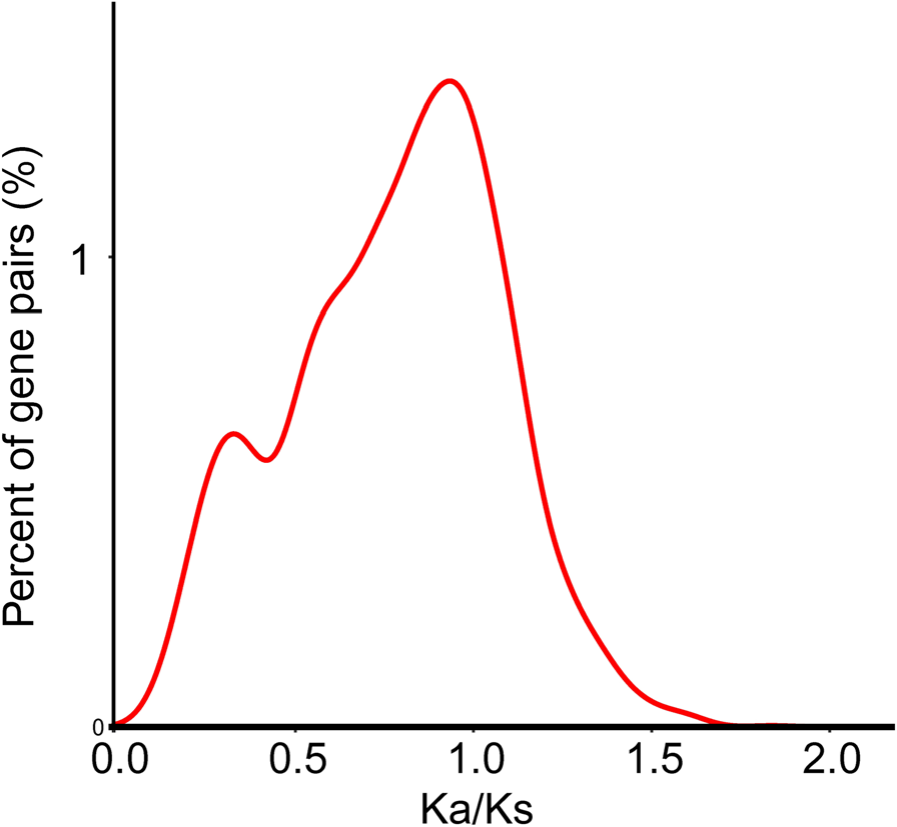
PTR genes nuderwent positive selection. X-axis indicates Ka/Ks ratio. Y-axis indicates PTR paralogous gene pairs. Ka/Ks>1 represents positive selection; Ka/Ks =1 represent neutral selection; Ka/Ks<1, represents purify selection

Whole genome duplications were detected using the synonymous mutation rate Ks. The rice genome experienced three genome duplication events (Fig. 4). The whole genome duplicationevent shared by gramineous plants occurred at ~ 96 million years ago [27]. After that, another two independent genomic duplications events occurred in rice [28, 29]. The Ka/Ks ratio reflects the extent to which all nucleotide sequences of a gene are positively selected during differentiation. If Ka/Ks is greater than 1, the gene is positively selected; if Ka/Ks is equal to 1, the gene is neutrally seleceted; if Ka/Ks is less than 1, the gene undergoes purify selection. The Ka/Ks ratios of paralogous PTR gene pairs were 0.08–1.83, and 693 gene pairs were greater than 1, indicating that many PTR genes underwent positive selection (Table S7, Fig. 5).

### Gene structure and motif composition of rice PTR genes

**Fig. 6:**
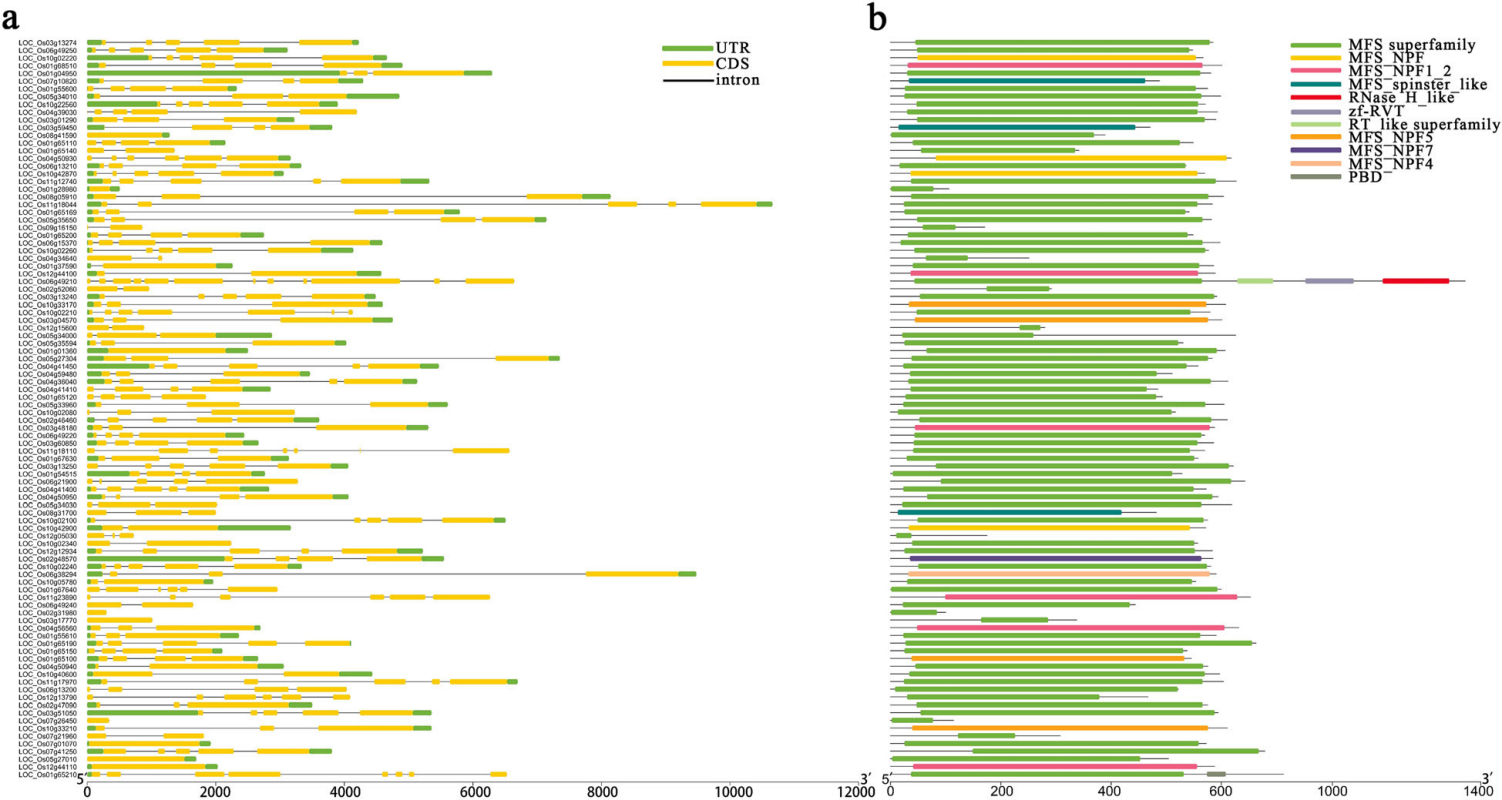
Gene structure and conserved motifs in PTR genes in rice. **a** Exon-intron structure of pineapple PTR genes. Green boxes indicate untranslated regions; yellow boxes indicate exons; black lines incicate introns. **b** The motif composition of rice PTR proteins. The eleven type motifs are displayed in different colored boxes. The information for each motif is shown on the right. The length of gene/protein can be estimated using the scale at the bottom

The information related to the evolution of the gene family can be obtained from gene structure analysis. Thus, we performed a gene structural analysis on the 96 PTR genes (Fig. 6a). In terms of the intron-exon composition, the PTR genes had 0–11 introns and 1–12 exons, and 10 genes contained only 1 exon. Usually, the structurally similar genes have closer evolutionary relationship.

Previous studies have shown that there are three highly conserved domains in most PTR genes [30]. In this study, there were five types of conserved domains in rice PTR genes, which were MFS, RNase_H_like, zf-RV, RT_like superfamily, and PBD. MFS can be further classified into MFS superfamily, MFS_NPF, MFS_NPF1_2, MFS_spinster_like, MFS_NPF5, MFS_NPF7, and MFS_NPF4. By performing conserved gene analysis on PTR genes, we found 77 genes had MFS superfamily domain and 16 genes contained NPF domain (Fig. 6b).

### The expression profiles of PTR genes in near isogenic lines

The expression profile of a gene is often related to its function. Previous studies have shown that the proteins encoded by PTR genes can transport nitrate, playing an important role in plant growth and development. Therefore, we used RNA-seq to analyze the expression profiles of 96 PTR genes in the near-isogenic rice lines NIL15 and NIL19 under HN and LN conditions.

The qualities of transcriptome sequencing and sequence alignment results are shown in Table S8. The cDNA library of 18 samples had high sequencing quality and good genome coverage, which was suitable for further analysis. Based on the gene expression levels in different samples, we performed correlation analysis on commonly expressed genes and differentially expressed genes (DEGs). The averaged correlation coefficient between biological replicates of the same sample was r = 0.9357 (Fig. S1), indicating good reproducibility and reliable experimental results.

We used the qRT-PCR method to validate the 18 expression genes identified from RNA-seq. The results showed that the qRT-PCR expression patterns of the 18 DEGs were consistent with RNA-seq analysis (Fig. S2), suggesting that the RNA-seq results were reliable for further analysis.

**Fig. 7:**
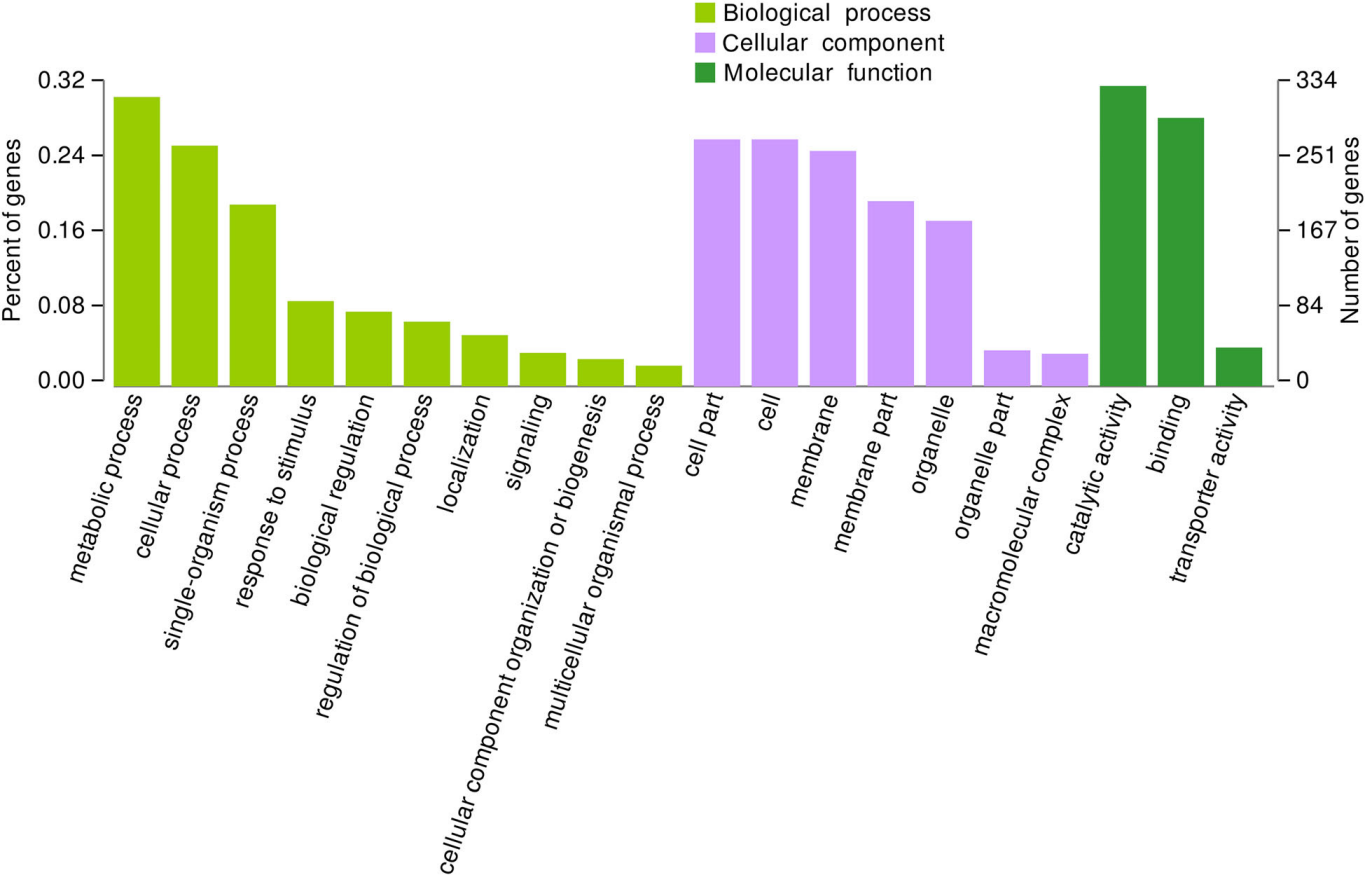
GO functional annotation for DEGs at three stages

Previous studies have shown that the differences between NIL15 and NIL19 are on chromosomes 6, 8, 9, and 10 [19]. These regions contain 6 PTR genes, which were *LOC_Os06g13200*, *LOC_Os06g13210*, L*OC_Os06g15370*, *LOC_Os06g21900*, *LOC_Os06g38294*, and *LOC_Os10g22560*. The transcriptome sequencing results showed that the number of DEGs between the two lines at three stages were 928, 1467 and 1586 (Fig. S3), respectively. After shifted to LN condition, the number of DEGs significantly increased first, and then stabilized. We also performed functional annotation on DEGs using the GO database (http://geneontology.org/), and the classification results are shown in Fig. 7. In the biological process category, metabolic process contained the most genes; in the cellular component category, there were more genes related to membrane or membrane components; in the molecular function category, transporter activity related genes showed up.

**Fig. 8:**
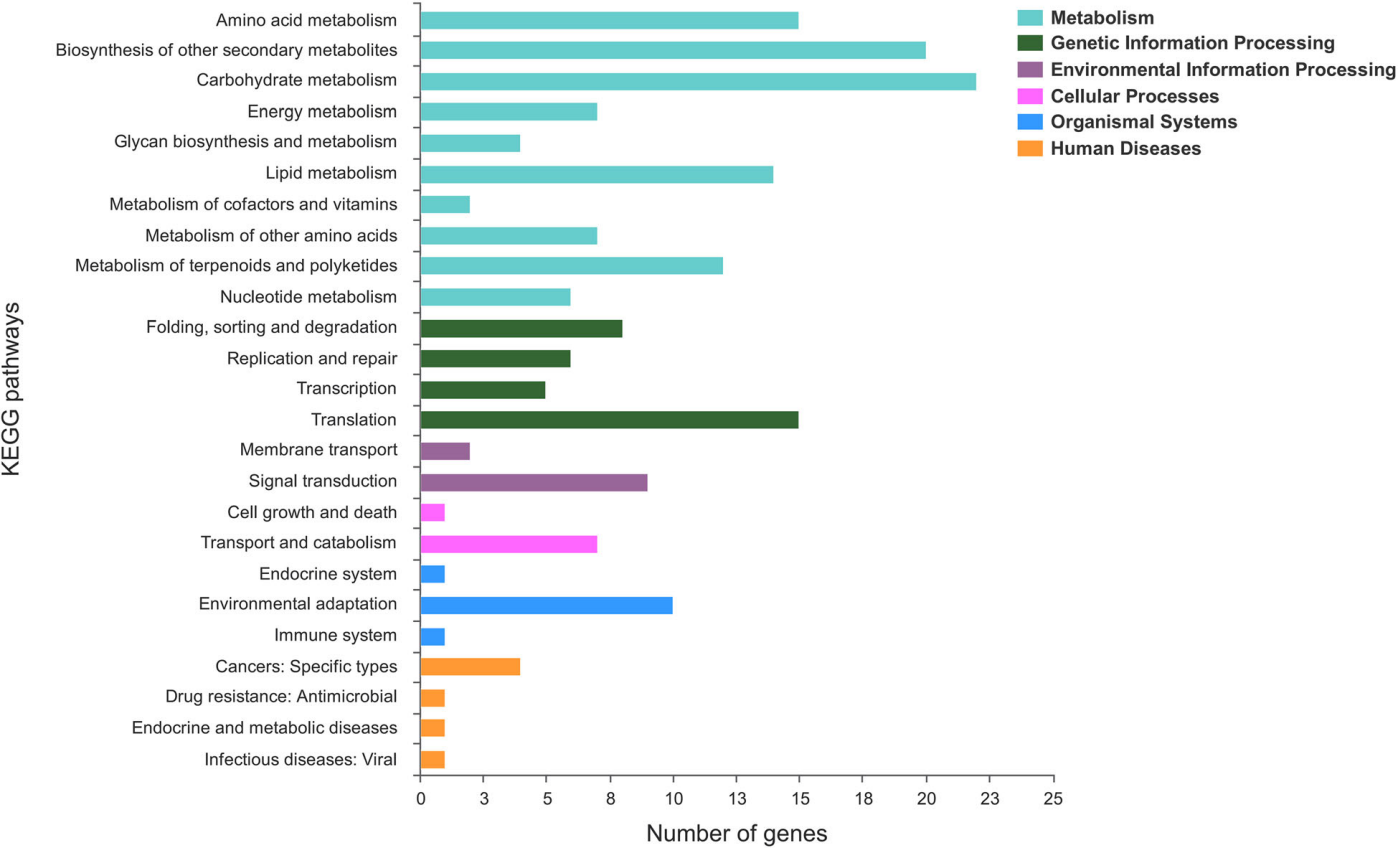
KEGG functional annotation for DEGs at three stages

In living organisms, different genes cooperate together to perform biological functions, and the same action across different genes form a pathway. We classified and annotated the DEGs using KEGG database (https://www.kegg.jp/), and the classification results are shown in Fig. 8. The main involved pathways were: metabolism, genetic information processing, environmental information processing, cellular processes, organismal systems and human diseases, in which the metabolism pathway contained the most genes. Also, N metabolism-related genes were included in energy metabolism group.

**Fig. 9:**
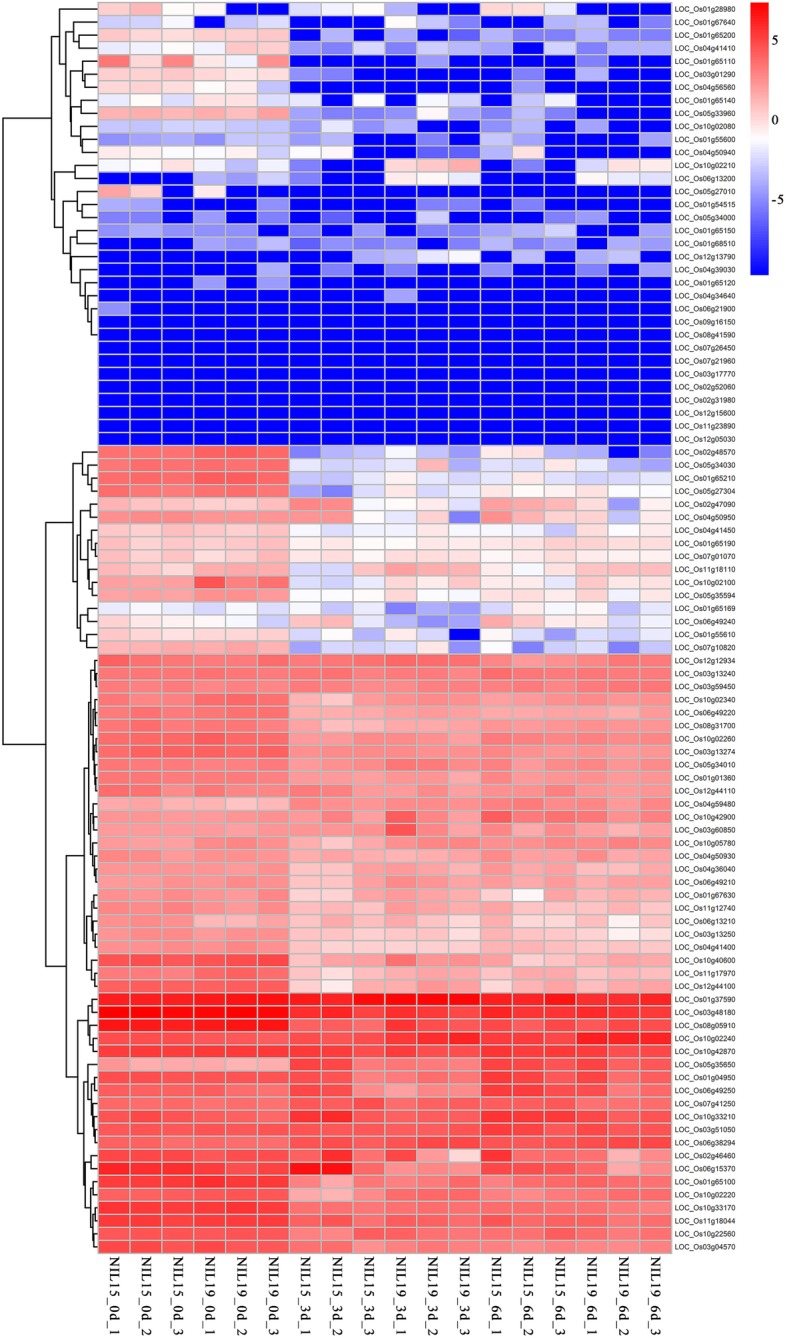
The expression profiles of 96 PTR genes at three stages in two near-isogenic lines. The cluster of PTR gene expression profiles are showed on the left; the gene name is showed on the right

The expression profiles of 96 PTR genes showed in Fig. 9. There were only 2 differentially expressed PTR genes between the two NILs at 0 d, which were *LOC_Os06g13210* and *LOC_Os10g02100*. However, at 3 d and 6 d, the number of differentially expressed PTR genes increased to 12 and 7 (Table S9), in which the *LOC_Os04g50950* (*OsPTR6*) [14], *LOC_Os06g49250* (*OsPTR9*) [15] and *LOC_Os11g12740* (*SP1*) [31] have been reported to participate in N uptake and transport in rice.

### The gene regulation network of NUE in rice

The expression profile data was analyzed using weighted gene co-expression network analysis (WGCNA) to predict the potential interaction between genes. The correlation coefficient of the gene expression levels was calculated and taken n-degree power, so that the numerical distribution of the correlation coefficient gradually followed scale-free distribution. 58,176 genes were screened, and the criteria were: (i) at least 50% of the samples are expressed the gene; (ii) remove the genes with the least variance change > = 25%. Finally, 20,282 genes were left for further analysis.

Then, we clustered the samples and found that the samples of 0 d, 3 d and 9 d could be well separated (Fig. S4a), indicating that the data quality was reliable enough for subsequent analysis. Then, a soft threshold was chosen to construct gene coexpression network (Fig. S4b,c). 20,282 genes were used for WGCNA analysis, and 400 genes were randomly selected from the gene set to draw the expression cluster heatmap (Fig. S5). We classified the genes with similar expression pattern into one module, and identified 25 modules. We analyze the gene features in each module and found the modules with biological significance (Fig. S6). Subsequently, GO and KEGG databases were used for functional enrichment analysis, and the blue, browen and turquoise modules were found to participate in N metabolism. The blue module contained six NUE-related genes: *LOC_Os01g36720* [32], *LOC_Os03g62200* [33], *LOC_Os04g40410* [34], *LOC_Os04g43070* [35], *LOC_Os05g39240* [36] and *LOC_Os06g49250* [15]. The brown module contained *LOC_Os01g61510* [33], *LOC_Os01g65000* [33], *LOC_Os02g47090* [16] and *LOC_Os04g50950* [14]. The turquoise module contained *LOC_Os02g02190* [34], *LOC_Os02g40710* [37], *LOC_Os02g40730* [38], *LOC_Os03g13274* [6], *LOC_Os03g48180* [39], *LOC_Os10g40600* [12, 40], *LOC_Os11g12740* [31] and *LOC_Os12g44100* [13].

**Fig. 10:**
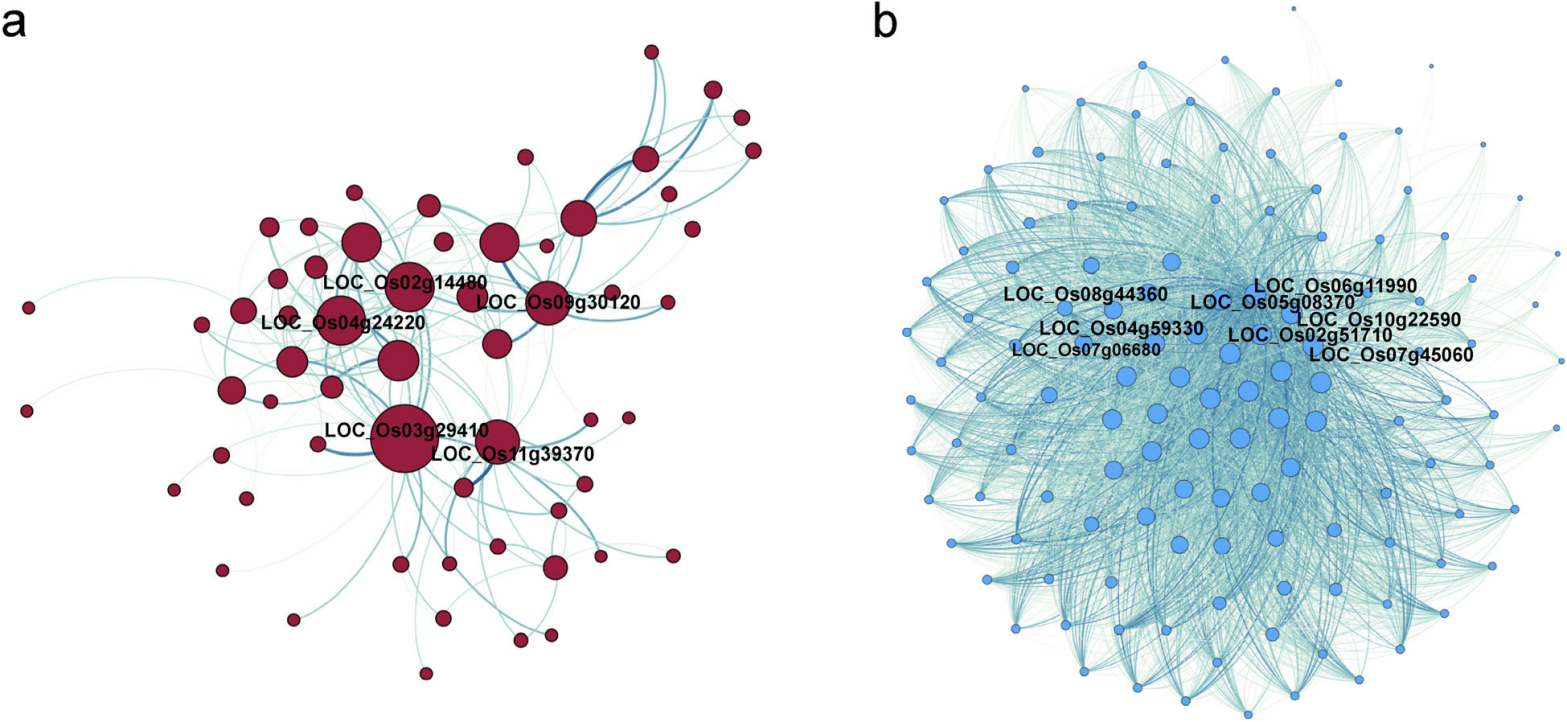
Gene co-expression networks. **a** 59 DEGs with the highest weight are in the brown module. **b** 129 differentially expressed PTR genes with the highest weight are in the turquoise module. The top most highly connected genes were signed. The red and blue circles represent DEGs

We performed WGCNA on these three modular genes. There were 59 DEGs in the brown module, and *LOC_Os03g29410*, *LOC_Os02g14480*, *LOC_Os04g24220*, *LOC_Os11g39370*, *LOC_Os09g30120* were hub genes in the regulatory network (Table S10, Fig. 10a). The functions of the most connected genes *LOC_Os03g29410*, *LOC_Os04g24220*, *LOC_Os02g14480*, and *LOC_Os11g39370* are related to protein kinase. Based on GO annotation, the functions of these genes were related to activity (GO: 0016301), stress (GO:0006950), biotic stimulus (GO: 0009607), signal transduction (GO: 0007165), and metabolic process (GO: 0008152). There were 129 DEGs in the turquoise module, and *LOC_Os06g11990*, *LOC_Os10g22590*, *LOC_Os08g44360*, *LOC_Os07g45060*, *LOC_Os07g06680*, *LOC_Os05g08370*, *LOC_Os04g59330* and *LOC_Os02g51710* were hub gene (Table S11, Fig. 10b). Due to the small number of DEGs in the blue module, we were not able to identify the hub genes.

## Discussion

In this study, we identified 96 PTR genes in Nipponbare genome, which was different from the 84 PTR genes identified by Zhao et al [30] The discrepancy was possibly due to: (i) with the development of sequencing technology, the Nipponbare genome database has been continuously edited and improved [41, 42]; (ii) the methods used in the two studies are different. Zhao et al. used BLASTP and BLASTN to search for PTR genes based on the conserved amino acid sequence of PTR domain. Our study used HMMER3 to search for PTR genes based on the conserved DNA sequence of PTR gene family, followed by manual verification.

The 96 PTR genes in rice can be classified into 5 groups, and the number of each group was not same. In addition, *LOC_Os03g13250* and *LOC_Os03g13274* (*OsNRT1*) [6], *LOC_Os03g01290* and *LOC_Os10g40600* (*OsNRT1.1B*) [11], *LOC_Os07g21960* and *LOC_Os12g44100* (*OsPTR2*) [13], *LOC_Os04g50940* and *LOC_Os04g50950* (*OsPTR6*) [14], *LOC_Os04g36040* and *LOC_Os11g12740* (*SP1*) [31] were more closely related. Therefore, these 5 genes may participate in N uptake and transport in rice.

Among the Asian cultivated rice, *japonica* rice had more PTR genes than *indica* rice; also, the Asian cultivated rice had more PTR genes than African cultivated rice. Although Nipponbare, R498, and *Oryza glaberrima* contained different numbers of PTR genes, their distributions in the 8 groups were evolutionarily consistent. When comparing rice with maize and sorghum, rice had the smallest genome but contained the most PTR genes. Leguminous plants have rhizobium on the roots, which can convert inorganic N in the air into organic N. We found that peanuts and soybeans had more PTR genes, which may be related to the need of N transport after N fixation. In the phylogenetic relationship, the distribution of PTR genes in monocots and dicots were significantly different, which may be because the two types of plants produced their own unique PTR genes during evolution [43].

Gene family can be formed by whole genome duplication or polyploidization, tandem duplication, and segmental duplication. In this study, we found that many PTR genes formed by tandem duplication were often closely arranged on the same chromosome. Previous studies have also shown that the genes in the same gene cluster have similar sequences and functions [44]. By using the PTR gene to analyze segmental duplication events, we found that there were five duplicated blocks in rice genome, which is consistent with the previous results [28]. Among the 96 PTR genes in rice, 25 were contained within the segmental duplication. Moreover, rice underwent three whole genome duplication events: the first whole genome duplication event shared by all gramineous plants, and the other two chromosomal fragments doubling events occurred independently on rice genome. This result is consistent with previous studies [28, 29, 45]. In addition, the PTR genes in the duplication region were clustered into the same group, with closer phylogenetic relationship. There were many PTR gene pairs with Ka/Ks values greater than 1, indicating that non-synonymous mutations led to functional changes in PTR gene, making it more suitable for the environmental change [46].

Gene structure is related its function. Previous studies have shown that there are three conserved motifs in the protein sequences of rice PTR genes [30]; and all of them are contained in MFS family, which has 12 transmembrane domains [47]. In this study, we found most PTR genes contained MFS family motifs, and the known rice NUE gene, *OsNRT1* [6], *OsNRT1.1A* [10],.*OsNRT1.1b* [8], *OsPTR2* [13], *OsPTR6* [14], *OsPTR9* [15], *OsNPF7.2* [20] and *SP1* [21] all contains MFS family domain. Moreover, 16 out of 96 PTR genes have NPF domain. In plants, NPF proteins transport a variety of substrates: nitrates, peptides, amino acids, etc [7] Due to the long intron of *LOC_Os11g18044,* the sequence length (10.7 kb) was greater than other PTR genes in rice; moreover, it contained a longer MFS family domain, suggesting that the function of this gene might be more complex.

Transcriptome analysis of the N15 and NIL19 revealed that most of the DEGs are associated with metabolism and transport activation, which is consistent with plant N metabolism pathways. There were only 2 PTR genes that were differentially expressed under HN condition, but 12 PTR genes changed expression level under LN condition. These results indicated that the PTR genes may play an important role in N metabolism in a cooperative way. Previous studies have shown that *LOC_Os06g49250* (*OsPTR9*) [15], *LOC_Os04g50950* (*OsPTR6*) [14], *LOC_Os11g12740* (SP1) [31] affect rice NUE, and these genes were all differentially expressed under LN condition.

In the three modules that may be associated with rice NUE, each module had different numbers of genes involved in N metabolism, but they all contained the genes related to the uptake and utilization of ammonium and nitrate, indicating the synergistic expression of ammonium and nitrate genes. This result suggested that N uptake, transport, assimilation and signal transduction involved complex gene regulatory networks in rice [5].

In the brown module, the functional annotation of hub genes were protein kinase. Previous studies have shown that some protein kinases are related to N metabolism in *Arabidopsis*. AtCIPK8, a calcineurin B-like (CBL)-interacting protein kinase (*CIPK*), was found to be participated in early nitrate signaling [48]*.* CIPK and mitogen-activated kinase kinase kinase (MEKK) are putative regulatory proteins involved in the early nitrate signalling [49]. Ca^2+^-sensor protein kinases (CPKs) are master regulators that orchestrate primary nitrate responses [50]. Furthermore, brassinosteroid (BR) signal kinase BSK3 regulates root elongation under limited N conditions [51]. A number of protein kinases have been identified in rice, mainly Calcium-dependent protein kinase, mitogen-activated protein kinase, stress-activated protein kinase. They play important roles in rice growth and development, biotic stress, abiotic stress, and expression regulation. Recently, researchers have found that multiple protein genes are related to NUE in rice. OsCPK12 participates in the response signal pathway in low Nitrogen stress, and overexpression of OsCPK12 enhances rice growth under low N conditions [52]. Hsieh et al. [53] found that the N-regulated genes, *Os02g0120100*, *Os02g0807000* and *Os06g0692600* etc., which encode transcription factors, protein kinases and protein phosphatases and may be involved in the regulation of early −N responses in rice roots. The serine/threonine/tyrosine (STY) protein kinase, ACTPK1, enhances ammonium uptake and use, and promotes growth of rice seedlings under sufficient ammonium [54]. *A calcium-dependent protein kinase* gene, *esl4*, which may function upstream of N-metabolism genes [55]. The overexpression of *Calcineurin B-like interacting protein kinase 2* gene, *OsCIPK2*, could increase N use efficiency in rice [56]. OsSAPK8 is a counterpart of AtOST1 in the activation of OsSLAC1, which is a nitrate-selective anion channel in rice [57]. *LOC_Os11g39370* is a BRASSINOSTROID INNOVATIVE 1-associated receiver kinase 1 precursor gene. Therefore, the brown module may be related to the regulation of N uptake and utilization in rice. The candidate gene *OsPTR10* identified in the previous study was also included in the brown module, and its function might be related to N metabolism.

## Conclusions

In this study, we comprehensively analyzed the PTR gene family in rice. 96 PTR genes were identification and classified into 5 main groups in Nippobare genome. The distribution of PTR genes between monocots and dicots was different, and legumes had a greater number of PTR genes. The rice genome experienced three genomic duplication events, and 25 rice PTR genes locate on 5 large segmental duplication regions. The Ka/Ks ratio indicated that many PTR genes had undergone positive selection. The structure and motif analysis revealed five types of conserved domains in rice PTR genes. The differentially expressed PTR genes increased significantly under LN conditions. Using WGCNA, we found three gene modules associated with NUE, especially the yellow module. These results provide information for a better understanding of the biological function for the PTR gene and will contribute to the genetic improvement of NUE in rice.

## Methods

### Genome sequence retrieval and PTR gene identification

The genome sequences of six species were downloaded from the following database: Nipponbare (MSU_7.0, http://rice.plantbiology.msu.edu/index.shtml), R498 [58] (CANU, http://www.mbkbase.org/R498/), *Oryza glaberrima* [59] (Oryza_glaberrima_V1, http://peanutgr.fafu.edu.cn/Genome_Browse.php), *Arabidopsis* [23] (TAIR10, http://plants.ensembl.org/Arabidopsis_thaliana/Info/Index), *Arachis hypogaea* [21] (PGR, http://peanutgr.fafu.edu.cn/Genome Browse.php), *Zea mays* [24] (B73_RefGen_v4, http://plants.ensembl.org/Zea_mays/Info/Index), *Glycine max* [22] (Glycine_max_v2.1, http://plants.ensembl.org/Glycine_max/Info/Index), and *Sorghum bicolor* [25] (Sorghum_bicolor_NCBIv3, http://plants.ensembl.org/Sorghum_bicolor/Info/Index). The PTR gene family HMM (hidden markov model) file was downloaded from the Pfam (http://pfam.xfam.org/). We did a genome-wide search on PTR genes using HMMER3, with e-value cut off = 0.001 and alignment sequence greater than 198 (50% of 395). The protein sequences were extracted and put into the three major databases: CDD (https://www.ncbi.nlm.nih.gov/Structure/cdd/wrpsb.cgi), SMART (http://smart.embl.de/), and PFAM (http://pfam.xfam.org/). Then, the resulting homologous family genes were manually verified for domain conservation. If conserved domain was detected in one of the three databases, it was considered as reliable family gene. Finally, the conserved domains and protein sequences were extracted.

### Phylogenetic tree construction

ClustalW was used to perform multi-sequence alignment on the identified PTR genes. The NJ tree was constructed using MEGA7, with the bootstrap repeat number of 1000 and other parameters as default. The phylogenetic tree was colored by iTOL (https://itol.embl.de/).

### Chromosomal distribution and gene duplication

All-against-all protein sequence alignment was performed using BLAST (basic local alignment search tool), and the protein pair with e-value < 0.00001, identity > 90%, and minimum coverage of matching region on query sequence to subject sequence > 75% were extracted as homologous protein. The homologous protein file and the protein coordinate file were input into MCScanX (http://chibba.pgml.uga.edu/mcscan2/) for collinear region identification. Finally, the collinear results were presented using Circos (http://circos.ca/).

### Analysis of gene structure and conserved motif

The PTR gene structure, mainly including UTR (untranslated region), introns and exons, was analyzed through GSDS (http://gsds.cbi.pku.edu.cn/). The motif sequence prediction was performed via MEME (http://meme-suite.org/tools/meme) and the motif map was presented using TBtools.

### Plant materials and hydroponics

Y11 is wlid rice (*Oryza rufipogon*), GH99 is elitericevariety (*Oryza sativa* L. ssp. *indica*), these materials are derived from Guangxi. Since autumn of 2007, Since autumn of 2007, we have used Y11 as the donor and GH998 as the recipient to construct the near-isogenic lines (NILs, BC_4_F_6_).. The NUE of NIL-13B4 (low NUE) and GH998 (high NUE) was 3.64 and 39.08%, respectively [19]. In this study, we selected the two NILs, NIL15 (NIL-13B4) and NIL19 (GH998), as experimental materials. The evenly germinated seeds (NIL15 and NIL19) were selected and cultured in 96-well cultivation instrument with 1.4 mM NH_4_NO_3_ (high nitrogen, HN) nutrient solution. The nutrient solution was prepared according to the method in Yoshida et al [60] The nutrient solution was changed every 3 days, and the pH was adjusted to ~ 5.5 using MERS-NaOH. The seedlings were grown in a intelligent artificial climate chamber (TOP Instrument, Hangzhou, Zhejiang, China) controlled at 28 °C with supplemental light from 6:00 to 19:00 h (13 h light /11 h dark) until the 3-leaf stage. Then, at 3-leaf stage, the nutrient solution was changed with 0.14 mM NH_4_NO_3_ (low nitrogen, LN) solution, and samples were collected at 0 d, 3 d, and 6 d for RNA-seq and quantitative real-time PCR (qRT-PCR), with three biologically replicates for each sample.

### RNA extraction and sequencing

The RNA of each sample was extracted using Trizol method. The purity and integrity of each RNA sample were examined by agarose gel electrophoresis. The purity of DNA was checked by Nanodrop (Thermo Fisher Scientific, USA) (OD 260/280 ≈ 2.0). RNA concentration was quantified by Qubit (Thermo Fisher Scientific, USA) accurately, and the minimum concentration was 50 ng/ul.

mRNA was isolated from total RNA using the Oligo (dT) coated magnetic beads. Then, the mRNA was randomly fragmented into ~ 300 bp fragments by fragmentation buffer. With reverse transcriptase and a six-base random primer (random hexamers), the mRNA was subsequently reverse-transcribed into single-strand cDNA, which then formed into a stable double-stranded structure via two-strand synthesis. The constructed library was sequenced using Illumina NovaSeq 6000.

### Sequencing quality control and alignment

After quality-control filtering, the clean reads were compared with the reference genome (http://rice.plantbiology.msu.edu/index.shtml) to obtain the mapped reads for subsequent analysis. Sequence alignment was performed using TopHat2 [61]. The mapping rate was usually higher than 70% when the reference genome was completely annotated and the experiment was free of contamination.

### Quantitative real-time PCR

The expression level of 18 genes were measured by qRT-PCR. The primers were designed based on target gene sequence (Table S1), and *Actin3* was used as a reference [62]. All qRT-PCR assays were carried out in 96-well plates using qTOWER 2.2 Quantitative Real-Time PCR Thermal Cycler (Analytik Jena, Germany). The reaction system included: 10 μl of 2× TransStart SYBR Green Master Mix (Vazyme, Nanjing, Jiangsu, China), 1 μl of each primer, 1 μl of template cDNA, complemented by ddH2O to 20 μl. The cycle program for product amplification was as follows: 94 °C for 5 min followed by 40 cycles of 94 °C for 30 s (denaturation), 55 °C for 30 s (annealing), and 72 °C for 30 s (extension). Tripricates were set for each sample. When the reaction was completed, the melting curve was analyzed and specificity of the product was determined based on the melting curve. The relative gene expression level was calculated by reference to the 2^−ΔΔCt^ method [63].

### Differentially expressed gene analysis

According to the results of feature counts alignment to reference genome and the annotation file, the gene read counts for each sample were obtained, followed by FPKM (fragments per kiolbase million) conversion to obtain standardized gene expression levels. DESeq2 [64] was used to perform statistical analysis on raw counts. The default thresholds were: p-adjust < 0.05 and **|**log2FC**|** > = 1, which yielded the differentially expressed genes between two groups or two samples which expression folds greater than 2 and *P* value less than 0.05.

### Weighted gene co-expression network analysis

After background correction and normalization of gene expression data, the nonstandard genes and less altered genes were filtered, so that the gene correlation intensity fell into scale-free distribution. After data pre-processing, the genes were classified: the genes with similar expression patterns were classified into one type, which was called a module. Then, the relationships among inter-module genes or intra-module genes were analyzed. Key modules were obtained by correlating with phenotypic data, and the module’s hub gene was obtained by visualization network analysis.

## Supplementary information

**Additional file 1 Table S1.** Primer sequences used for qRT-PCR.

**Additional file 2 Table S2.** Identification of 96 PTR genes in Nipponbare.

**Additional file 3 Table S3.** Identification of 85 PTR genes in R498.

**Additional file 4 Table S4.** Identification of 78 PTR genes in *Oryza glaberrima*.

**Additional file 5 Table S5.** The distribution of PTR gene in Nipponbare, R498 and *Oryza glaberrima*.

**Additional file 6 Table S6.** The distribution of PTR genes in six species.

**Additional file 7 Table S7.** Ka/Ks ratio of PTR gene pairs in rice.

**Additional file 8 Table S8.** Sequencing data and mapping to Nipponbare genome.

**Additional file 9 Table S9.** The differentially expressed PTR genes at three stages.

**Additional file 10 Table S10.** The differentially expressed genes in the brown module.

**Additional file 11 Table S11.** The differentially expressed genes in the turquoise module.

**Additional file 12 Fig. S1.** The correlation coefficient of 18 samples based on the gene expression levels. The colored box shows correlation coefficient.

**Additional file 13 Fig. S2.** The qRT-PCR was used to validate the 18 expression genes identified from RNA-seq. X-axis represents the stage of 3 treatments, the purple column represents qRT-PCR results in NIL19, the green column represents qRT-PCR results in NIL15, the blue column represents RNA-seq results in NIL19, and the yellow column represents RNA-seq results in NIL15. Y-axis represents the relative level of gene expression, qRT-PCR uses 2^−ΔΔCt^ value, and RNA-seq uses FPKM value. Error bars indicate standard deviations of three biological repetitions.

**Additional file 14.** Fig. S3. The DEGs were screened by DESeq2. A1 vs A2 represents 928 DEGs btween NIL15 and NIL19 at 0 d. B1 vs B2 represents 1467 DEGs btween NIL15 and NIL19 at 3 d. C1 vs C2 represents 1586 DEGs btween NIL15 and NIL19 at 6 d.

**Additional file 15 Fig. S4.** Sample cluster dendrogram and soft-thresholding (β) values. **a** Sample cluster dendrogram and clinical trait heatmap of 18 samples based on their expression profile. **b** Analysis of scale-free fit index of each β value from 1 to 20. **c** Analysis of mean connectivity of each β value from 1 to 20. β =10 was chosen for subsequent analyses as it has the biggest mean connectivity when the scale-free fit index is up to 0.895.

**Additional file 16 Fig. S5.** The heat-map of the gene network of 400 randomly selectedgenes. The gene dendrogram and module assignment are also shown along the top. Color scale: yellow indicates low correlation, and red indicates high correlation.

**Additional file 17 Fig. S6.** Identification of modules associated with NUE in two near-isogenic lines. Color scale: yellow indicates low correlation, and red indicates correlation.

## Data Availability

All data analysed in this study are included in the main manuscript and its additional files. The data supporting the findings of this work are available in the paper and its supplementary Information files. The RNA-seq data that support the findings of this study have been deposited to National Center for Biotechnology Information (NCBI) Sequence Read Archive (SRA) with the accession code PRJNA573824 [https://www.ncbi.nlm.nih.gov/bioproject/PRJNA573824].

## Abbreviations

N: Nitrogen
PTR: Peptide transporter
NUE: Nitrogen use efficiency
NPF: NRT1/PTR family
DEG: Differentially expressed genes
NIL: Near-isogenic line
HMM: Hidden markov model
NJ: Neighbor-joiningmethod
UTR: Untranslated region
qRT-PCR: Quantitative RT-PCR
FPKM: Fragments per kiolbase million
GO: Gene ontology
KEGG: Kyoto encyclopedia of genes and genomes
WGCNA: Weighted gene co-expression network analysis
