# Supplementary figures and images for: Genome-wide identification of the peptide transporter family in rice and analysis of the PTR expression modulation in two near-isogenic lines with different nitrogen use efficiency
Source: BMC Plant Biol. 2020 May 6;20:193. doi: 10.1186/s12870-020-02419-y (PMC7203820; doi:10.1186/s12870-020-02419-y)

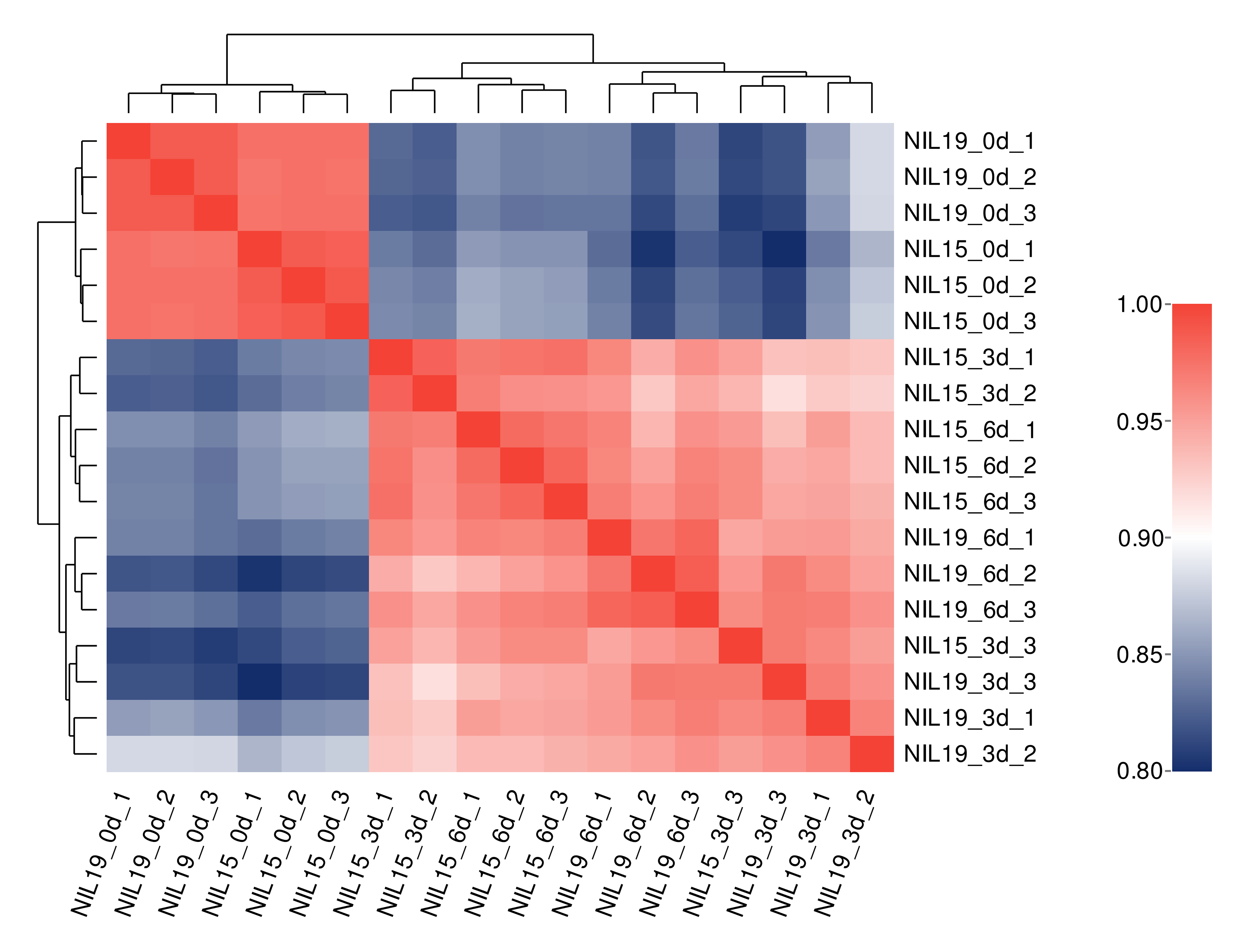

Supplement: Supplementary file 12 — Additional file 12 Fig. S1. The correlation coefficient of 18 samples based on the gene expression levels. The colored box shows correlation coefficient. [file 12870_2020_2419_MOESM12_ESM.jpg]

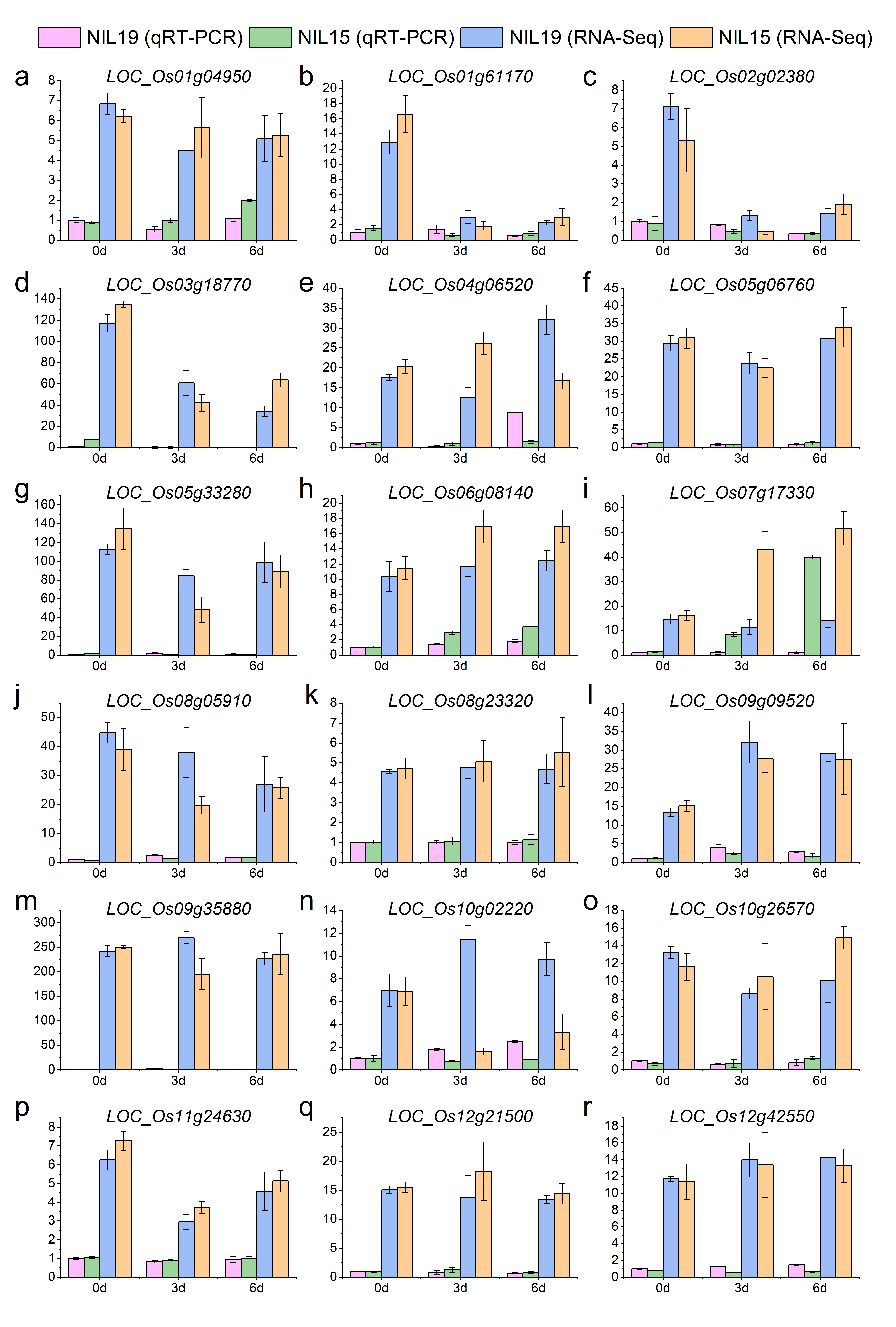

Supplement: Supplementary file 13 — Additional file 13 Fig. S2. The qRT-PCR was used to validate the 18 expression genes identified from RNA-seq. X-axis represents the stage of 3 treatments, the purple column represents qRT-PCR results in NIL19, the green column represents qRT-PCR results in NIL15, the blue column represents RNA-seq results in NIL19, and the yellow column represents RNA-seq results in NIL15. Y-axis represents the relative level of gene expression, qRT-PCR uses 2−ΔΔCt value, and RNA-seq uses FPKM value. Error bars indicate standard deviations of three biological repetitions. [file 12870_2020_2419_MOESM13_ESM.jpg]

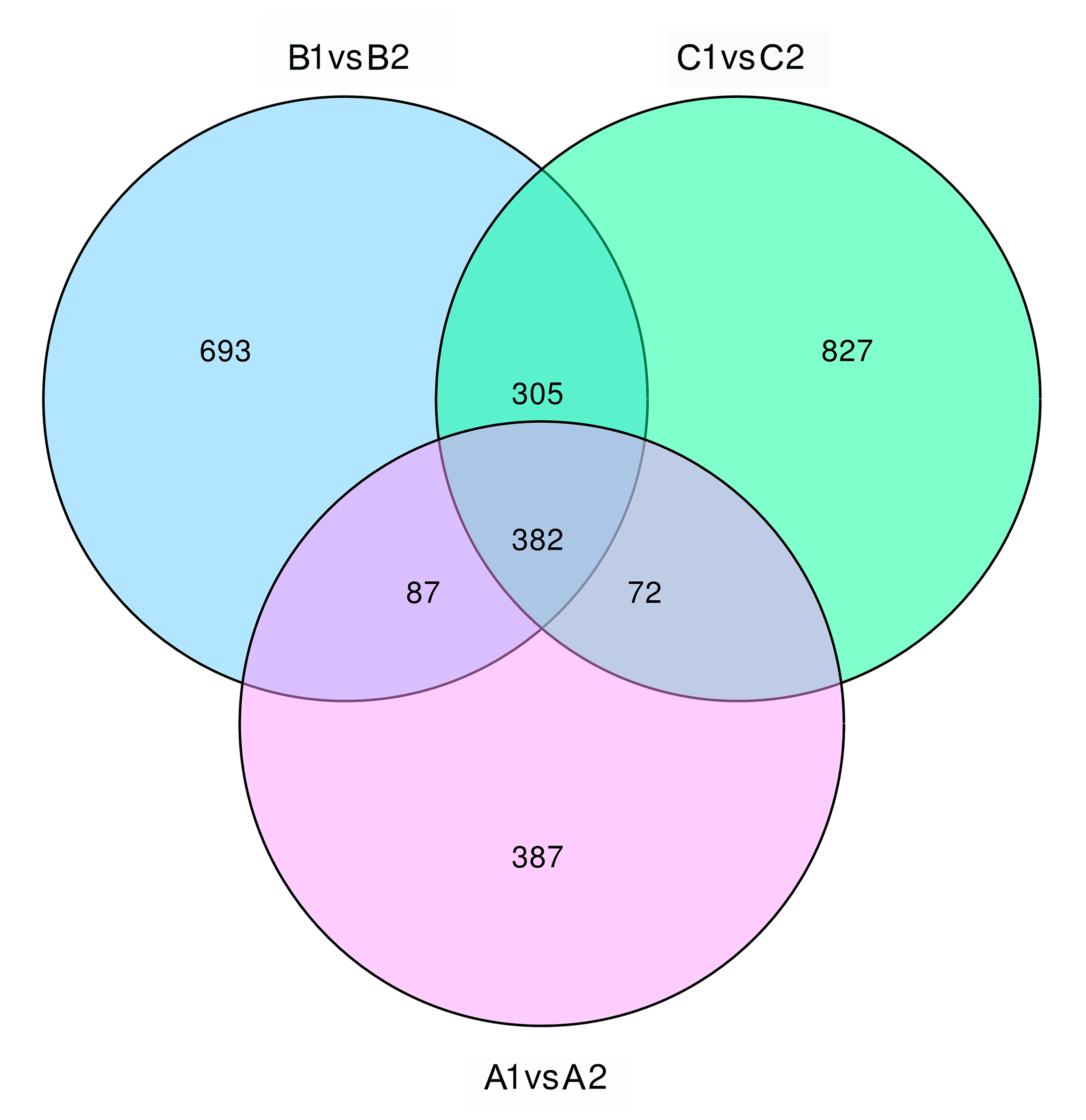

Supplement: Supplementary file 14 — Additional file 14. Fig. S3. The DEGs were screened by DESeq2. A1 vs A2 represents 928 DEGs btween NIL15 and NIL19 at 0 d. B1 vs B2 represents 1467 DEGs btween NIL15 and NIL19 at 3 d. C1 vs C2 represents 1586 DEGs btween NIL15 and NIL19 at 6 d. [file 12870_2020_2419_MOESM14_ESM.jpg]

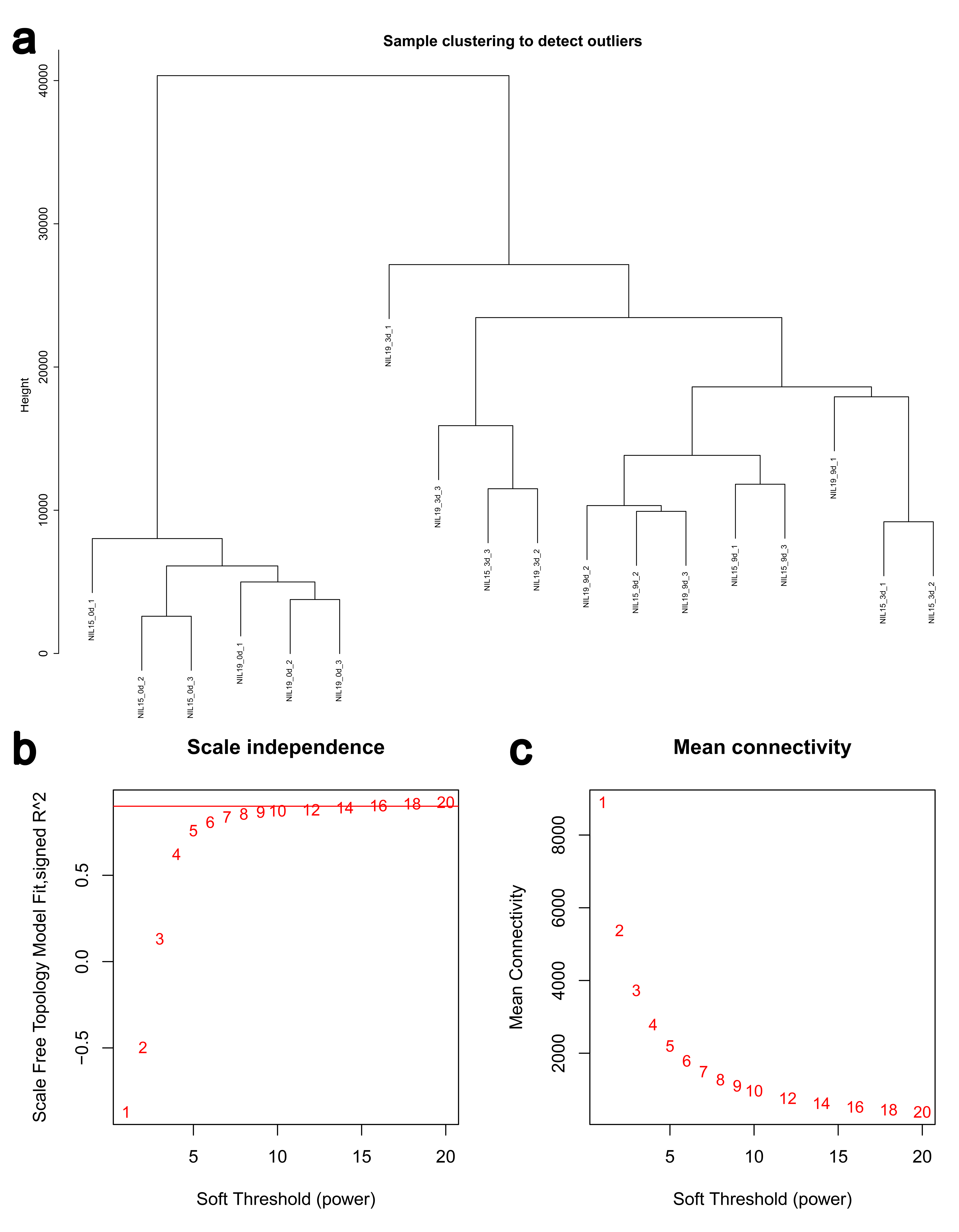

Supplement: Supplementary file 15 — Additional file 15 Fig. S4. Sample cluster dendrogram and soft-thresholding (β) values. a Sample cluster dendrogram and clinical trait heatmap of 18 samples based on their expression profile. b Analysis of scale-free fit index of each β value from 1 to 20. c Analysis of mean connectivity of each β value from 1 to 20. β =10 was chosen for subsequent analyses as it has the biggest mean connectivity when the scale-free fit index is up to 0.895. [file 12870_2020_2419_MOESM15_ESM.jpg]

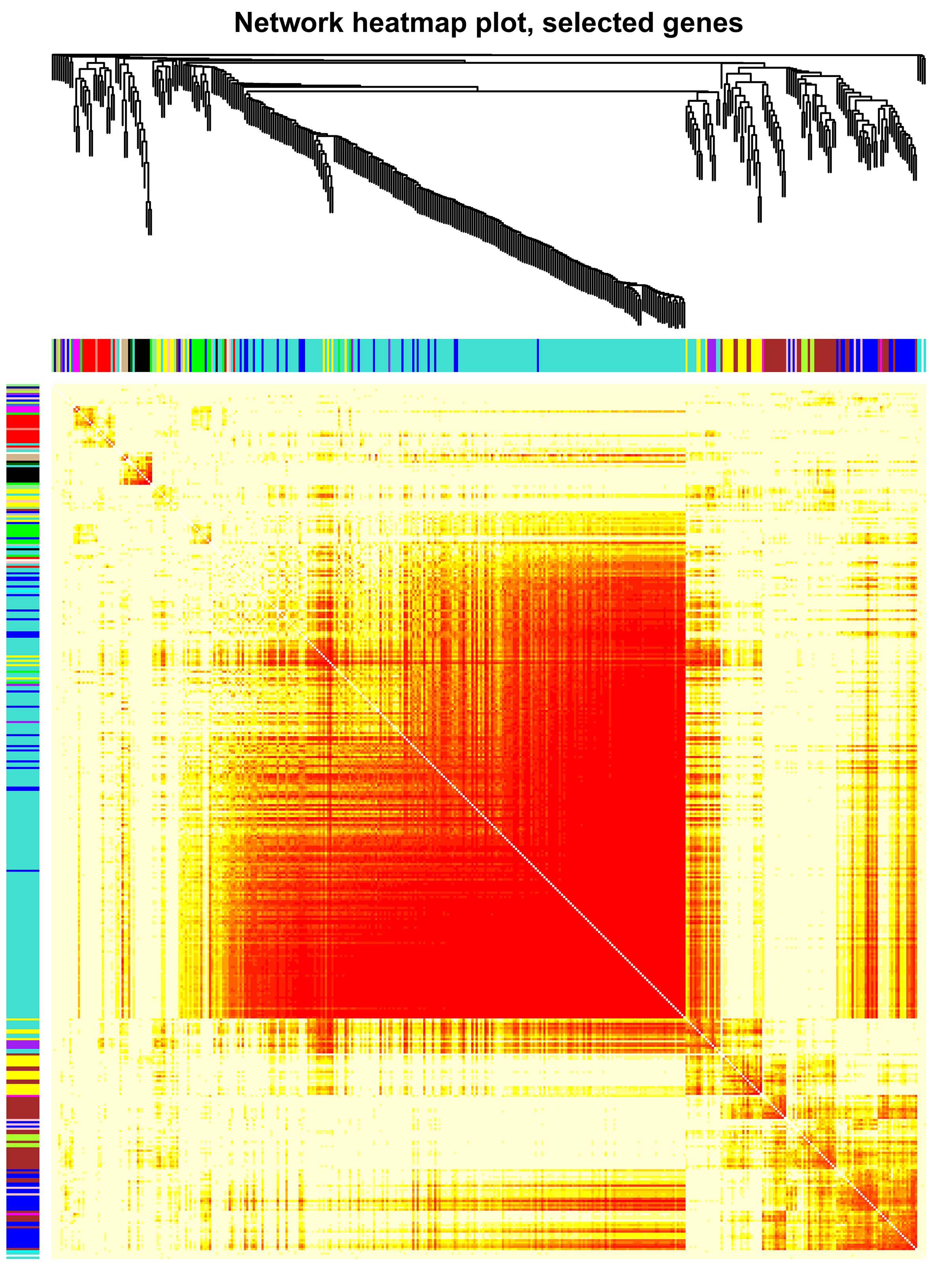

Supplement: Supplementary file 16 — Additional file 16 Fig. S5. The heat-map of the gene network of 400 randomly selectedgenes. The gene dendrogram and module assignment are also shown along the top. Color scale: yellow indicates low correlation, and red indicates high correlation. [file 12870_2020_2419_MOESM16_ESM.jpg]

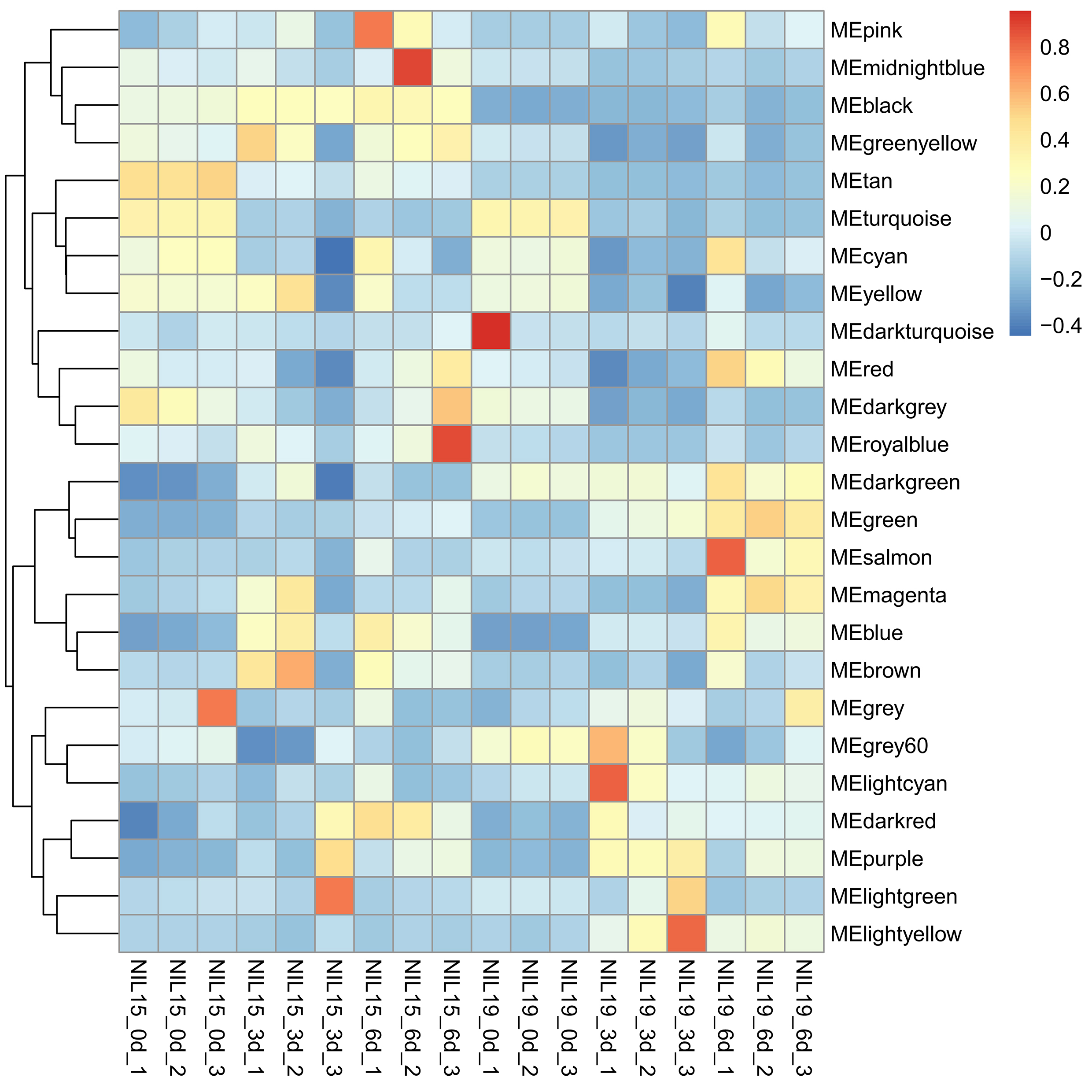

Supplement: Supplementary file 17 — Additional file 17 Fig. S6. Identification of modules associated with NUE in two near-isogenic lines. Color scale: yellow indicates low correlation, and red indicates correlation. [file 12870_2020_2419_MOESM17_ESM.jpg]
